# Effectiveness and toxicity of cetuximab with concurrent RT in locally advanced cutaneous squamous cell skin cancer: a case series

**DOI:** 10.18632/oncotarget.28470

**Published:** 2023-07-07

**Authors:** Mark Chang, Wolfram Samlowski, Raul Meoz

**Affiliations:** ^1^University Medical Center of Southern Nevada, Las Vegas, NV 89102, USA; ^2^Comprehensive Cancer Centers of Nevada, Las Vegas, NV 89148, USA; ^3^Department of Internal Medicine, University of Nevada, Las Vegas (UNLV), Las Vegas, NV 89102, USA; ^4^Department of Internal Medicine, University of Nevada, Reno, NV 89557, USA

**Keywords:** keratinocyte carcinoma, squamous cell skin cancer, cetuximab, epidermal growth factor receptor, radiation therapy

## Abstract

Background: Treatment for locally advanced cutaneous squamous cell cancers (laCSCC) remains poorly defined. Most laCSCC tumors express high levels of epidermal growth factor receptors (EGFR). Cetuximab has activity in other EGFR expressing cancers and enhances the effectiveness of radiotherapy.

Methods: A retrospective review of institutional data identified eighteen patients with laCSCC treated with cetuximab induction and concurrent radiotherapy. The loading dose of cetuximab was 400 mg/m² IV. Subsequent weekly doses of 250 mg/m² IV were infused throughout the period of radiation. The treatment doses ranged from 4500–7000 cGy, with a dose fraction of 200-250 cGy.

Results: The objective response rate was 83.2% with 55.5% complete responses and 27.7% partial responses. Median progression-free survival was 21.6 months. Progression-free survival was 61% at 1 year and 40% at 2 years. With longer follow-up, some patients developed a local recurrence (16.7%), distant metastases (11.1%) or a second primary cancer (16.3%). Cetuximab was well tolerated, with 68.4% patients experienced only mild acneiform skin rash or fatigue (Grade 1 or 2). Radiotherapy produced expected side effects (skin erythema, moist desquamation, mucositis).

Discussion: Cetuximab plus radiotherapy represents an active and tolerable treatment option for laCSCC, including patients with contraindications for checkpoint inhibitor therapy.

## INTRODUCTION

Basal cell and squamous cell (keratinocyte-derived) skin cancers are extremely common. It is estimated that the incidence is 3.3–5.4 million patients each year in the U.S [1]. Due to their frequency, these cancers are not reportable in the NCI Surveillance, Epidemiology, and End Results (SEER) cancer registry. The incidence of keratinocyte-derived skin cancers has increased steadily over decades [1].

Cutaneous squamous cell carcinoma (CSCC) represents approximately 20% of keratinocyte-derived skin cancers with an estimated 3–7% of CSCC patients developing recurrent invasive, regionally metastatic, or distant metastatic disease [2]. Precise data concerning the incidence of keratinocyte-derived skin cancers, as well as the number of patients with more advanced stage disease and their outcome are not readily available. Karia estimated that in 2012 between 5604 to 12,572 CSCC patients developed nodal metastasis (~4% of estimated CSCC patients), resulting in 3932 to 8791 deaths (~1.5% mortality) [3].

In general, CSCC is predominantly a disease of elderly, fair skinned, heavily sun-exposed Caucasians. Known risk factors for aggressive CSCC behavior include tumor-related factors, such as head and neck primary sites, indistinct infiltrative borders of the lesion, rapid growth, tumor diameter >2 cm, invasion to >2.0 mm depth, and perineural extension [2]. Host factors that are thought to predict more aggressive behavior include immunosuppression (e.g., organ transplantation, co-morbid conditions such as HIV, indolent lymphomas and CLL) and tumor recurrence after previous surgery or radiotherapy [4]. A number of staging systems have been defined to try to identify high risk individuals for more aggressive therapy and close monitoring [5]. These include the American Joint Commission for Cancer (AJCC), Union for International Cancer Control (UICC) and the Brigham and Women’s Hospital staging systems [6, 7]. In the Brigham and Women’s staging system, T2b or T3 tumors appear to be high-risk and have a greater than 20% risk of lymph node involvement [8, 9].

Treatment options for laCSCC remain poorly defined. In the past, after failure of the initial resection, radiotherapy with or without added chemotherapy was frequently utilized [2, 10, 11]. Since the average advanced CSCC patient is elderly, often with significant comorbid medical conditions, aggressive chemotherapy proved to be poorly tolerated.

The epidermal growth factor receptor (EGFR) is overexpressed in virtually all squamous cell skin cancers [12]. As single agents, EGFR inhibitor monoclonal antibodies cetuximab and panitumumab have shown modest efficacy in phase 2 clinical trials in patients with metastatic cutaneous squamous-cell carcinoma, but durable responses have been uncommon [13–16]. There is, however, an extensive literature concerning treatment squamous cell carcinoma of the head and neck (SCCHN) showing that the EGFR directed monoclonal antibody cetuximab plus radiotherapy has significant activity in this EGFR-driven cancer.

Since there was no established standard therapy for laCSCC patients in 2014, we began treating patients with bulky unresectable tumors with cetuximab and concurrent radiotherapy due to their potential synergy. This was reasonable because most of our patients had primary tumors involving head and neck primary sites. It should be noted, that while synergy of cetuximab plus radiotherapy has been demonstrated in SCCHN, formal evaluation of additive or synergistic benefit in CSCC has not yet been established versus radiotherapy alone. More recently, PD-1 directed monoclonal antibodies such as cemiplimab and pembrolizumab have received FDA approval for treatment of recurrent, unresectable, or metastatic CSCC [17, 18]. Unfortunately, even today, many patients are not candidates for treatment with PD-1 antibodies, due to organ transplants and underlying autoimmune conditions [19]. Thus, additional treatment options are needed.

We performed a retrospectively review of treatment outcome and toxicity in our patients who received concurrent cetuximab and radiotherapy to show an additional potentially effective treatment option for patients with laCSCC. The goal is also to provide data to inform the design of potential prospective clinical trials.

## RESULTS

Eighteen eligible patients were identified. Patient characteristics are shown (Table 1). Of the 18 patients identified, 16 were male (88.8%) and 2 were female (11.1%). The median age of patients was 76 ± 11 years (SD) with an age range of 47–92 years. Seventeen patients were Caucasian and 1 was African American.

**Table 1:** Patient characteristics and treatment

UPN	Age	Sex	Race	Primary Site	B&W Stage	Comorbidities	Doses cetuximab	Cetuximab duration (months)	Fx size (cGy)	# Fx	Total RT Dose (cGy)	Elapsed time (days)
1	60	M	C	Cheek	T2b, N0	Lung transplant	7	1.4	200	25	5000^*^	32
2	71	M	C	Pre-auricular skin	T2b, N0	Gout, arthritis, hypothyroid	10	2.6	200	33	6600^*^	64
3	91	M	C	Neck	III	Polio, arthritis, HTN, CAD	10	2.8		35	5000 1600 Boost	54
4	73	M	C	Scalp and parotid	III	COPD, DM, CAD, lung nodules, Afib, HTN	9	1.9	200	30	5000^*^ 1000 Boost	41
5	78	F	C	Scalp and neck	IVA	Dementia, CKD, Hypothyroidism	17	4.4	200	25	5000^*^	35
6	87	M	C	Scalp	T2b, N0	None	11	2.6	200	30	6000^*^	47
7	70	M	C	Scalp	III	Lung transplant	6	1.8	200	21	6000 4200 total scalp XRT	28
8	73	M	C	Rt Ankle Rt Groin	III	Melanoma, UC	6	1.4	200	25	5000 Rt^*^ ankle, 3800 Rt^*^ Groin	35
9	67	M	C	Scalp to nodes	III	None	7	1.7	200	35	7000 Parotid	58
10	82	M	C	Cheek	T2b, N0	Hypothyroidism, BPH	6	1.2	200	30	6000^*^	42
11	79	M	C	Scalp	T3, N0	COPD, CAD, HTN, Arthritis	5	0.9	200	21	4200	28
12	92	F	C	Eyebrow	T3, N0	Arthritis, DM, CAD, HTN, Breast Cancer	7	1.4	180	25	4500	34
13	76	M	C	Scalp	T2b, N0	NHL, DVT, gout	18	15.1	200	25 25 8 25	5000 Scalp^*^ 5000 Neck 1600 Boost 5000 Palliative	35 36 12 40
14	70	M	C	Ear	T2b, N0	Renal transplant, HTN, DM, CHF	4	0.8	200	25	5000 R Ear 6600 Temporal bone	35
15	76	M	C	Neck node	III	Asthma	9	12.6	200	35	5000 Neck 2000 Boost	51
16	74	M	C	Scalp	IVA	Heart transplant, bronchitis, arthritis, CKD	9	24.2	250	21 15	5250^*^ 3000	28 21
17	85	M	C	Scalp	T2b, N0	HTN, CAD, Afib, TIA	9	2.3	200	32	6400^*^	57
18	47	M	AA	Legs	T2b, N0	Gout	6	1.2	250	24	6000^*^	32

^*^Electron beam therapy. Abbreviations: UPN: unique patient number; M: male; F: female; C: Caucasian; AA: African American; B&W: Brigham and Womens; HTN: hypertension; CAD: coronary artery disease; COPD: chronic obstructive pulmonary disease; DM: diabetes mellitus; Afib: atrial fibrillation; CKD: chronic kidney disease; UC: ulcerative colitis; CA: cancer; BPH: benign prostatic hypertrophy; DVT: deep venous thrombosis; TIA: transient ischemic attack; FX: radiotherapy fraction; RT: radiotherapy.

The median number of cetuximab doses was 8.0 ± 3.7 (SD). The range was 4–18. The median cumulative dose of cetuximab was 4750 ± 2872 mg. The median duration of cetuximab therapy was 1.9 ± 6.2 months and ended with completion of radiotherapy.

**Figure 1:**
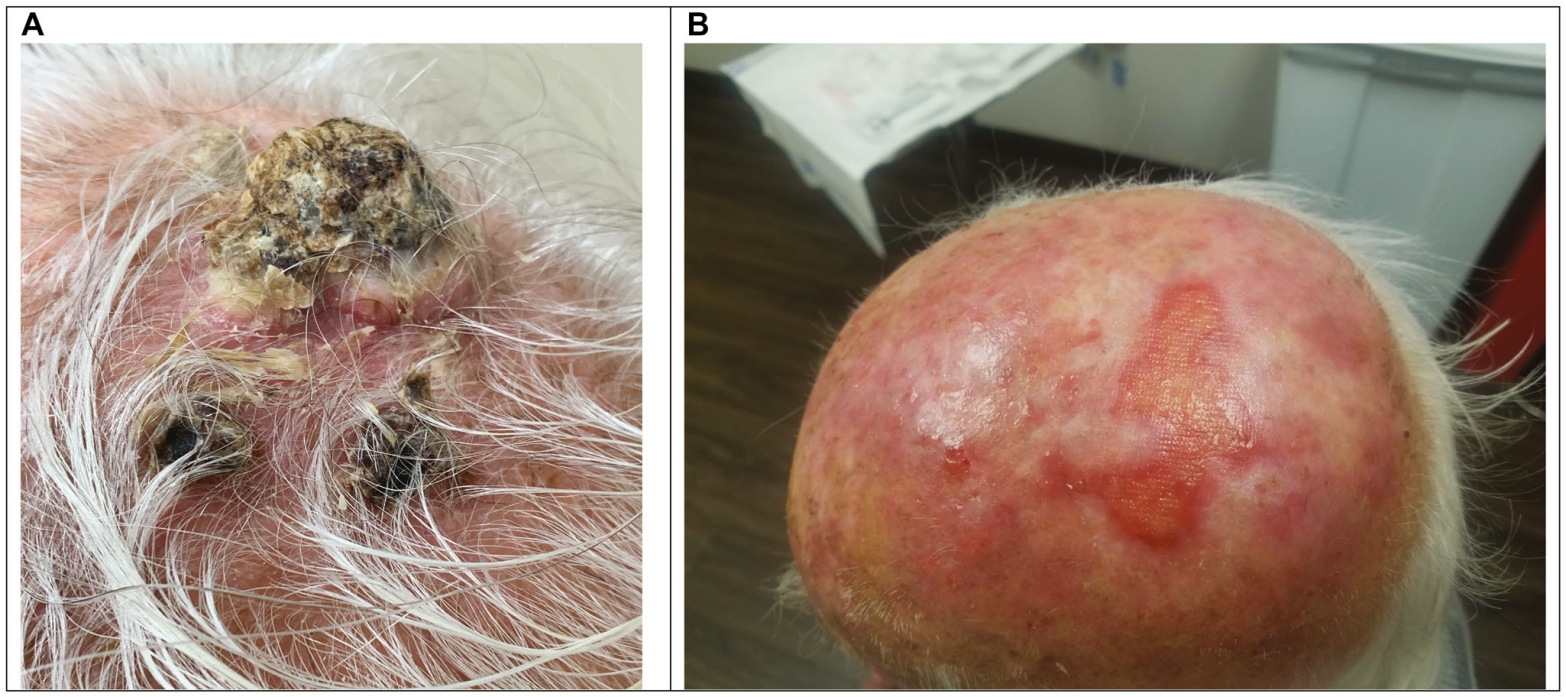
Example of treatment response. Patient 17 Pre-treatment scalp photograph (**A**). Patient 17 scalp photograph at 14 months post-treatment with Cetuximab/RT (**B**).

The response rates for concurrent therapy with cetuximab plus radiation therapy included 10 patients (55.5%) who achieved an objective complete response (CR), 4 (27.7%) had a partial response at the treated site (Table 2). Thus, the objective response rate (CR + PR) was 83.2%. As an example of a typical response is shown, (Figure 1) demonstrating dramatic remission of bulky scalp tumors, including in-transit metastases, following concurrent treatment. This patient achieved a durable complete response after treatment, which continues at over 2-year follow-up.

**Table 2:** Treatment outcome

UPN	Cetuxumab toxicity	RT toxicity	OR	PFS (months)	OS (months)	Current status	Cause of death
1	Acneiform Rash	Dermatitis and mucositis	PD	1.7	25.2	D-2nd primary	NSCLC
2	Acneiform Rash	Dermatitis	CR	17.2	17.2	A-NED	–
3	Acneiform Rash	Mucositis, moist desquamation	CR	19.7	19.7	A-NED	–
4	Acneiform Rash	Dermatitis	PR	6.2	12.2	D-other	COPD
5	None	Dermatitis	CR	17.9	17.9	A-NED	–
6	Fatigue, acneiform rash	Moist desquamation skin necrosis	CR	18.2	33.4	A-NED	–
7	Acneiform Rash	Dermatitis, mucositis.	PR	8.2	8.2	D-2nd primary	NSCLC
8	Acneiform Rash	Moist desquamation	PR	20.0	62.5	DOD	–
9	None	Moist desquamation with necrosis, fatigue, mucositis	PR	3.8	3.8	D-other	Suicide
10	Acneiform Rash	Mucositis. Auditory canal inflammation	CR	60.4	60.4	A-NED	–
11	Acneiform Rash	None	PD	0.9	3.5	DOD	Disease progression
12	Acneiform Rash	None	CR	5.6	5.6	D-2nd primary	Second primary
13	None	Dermatitis and mucositis	CR	60.7	60.7	D-2nd primary	Disease progression, second primary
14	Acneiform rash and dry eyes	Moist desquamation	PD	5.9	44.8	D-other	Disease progression
15	None	None	CR	69.9	69.9	A-NED	
16	SOB	dermatitis, photophobia	SD	21.6	45.8	DOD	disease progression
17	acneiform rash	Moist desquamation	CR	14.4	14.4	A-NED	–
18	None	dermatitis	CR	60.3	60.3	A-NED	–

Abbreviations: UPN: unique patient number; CR: complete response; PR: partial response; SD: stable disease; PD: progressive disease; PFS: progression free survival; OS: overall survival; D-2nd primary: died of second primary cancer; A-NED: alive: no evidence of disease; D-other: died of non-cancer related causes; DOD: died of CSCC; NSCLC: non-small cell lung cancer; COPD: chronic obstructive pulmonary disease.

**Figure 2:**
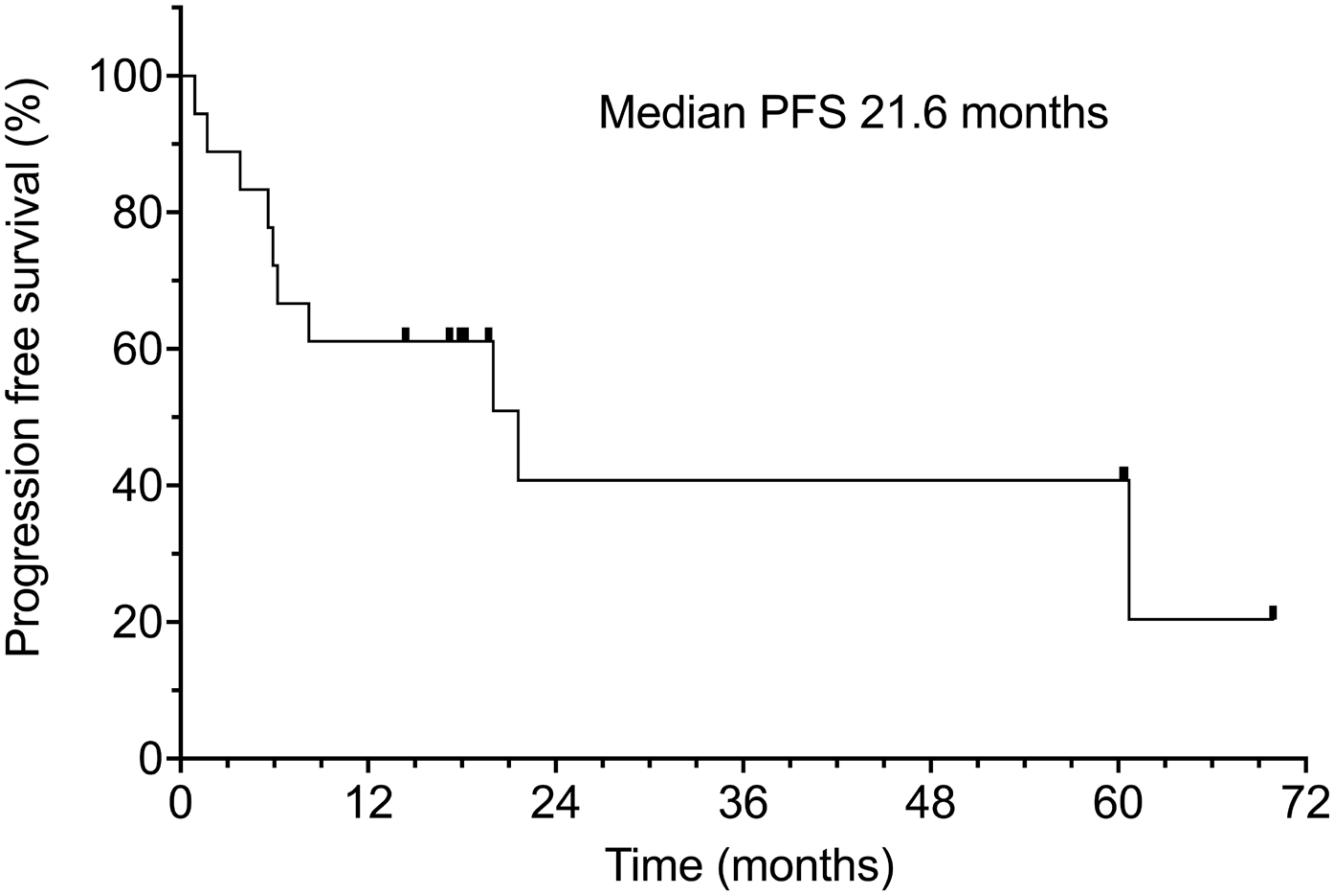
Progression-free survival of all 18 patients. Censored patients are indicated by hash marks.

Median progression free survival was 21.6 months at a median follow-up of 18 months (range of 0.9–70 months) (Table 2). The apparent drop-off in the PFS graph after 21 months is due to the diminishing number of patients at risk for progression. Only one patient eventually relapsed within the treatment site (5.5%), 2 relapsed with marginal recurrences at the edge of the irradiated field (11.1%), 3 progressed in regional lymph nodes or in-transit metastases (16.6%). With longer follow-up, 2 patients developed distant metastases (11.1%) and 3 patients developed aggressive 2nd primary neoplasms (16.6%) (one of these also had a concurrent regional recurrence of CSCC). Progression-free survival was 61% at 1 year and 40% at 2 years. (Figure 2).

**Figure 3:**
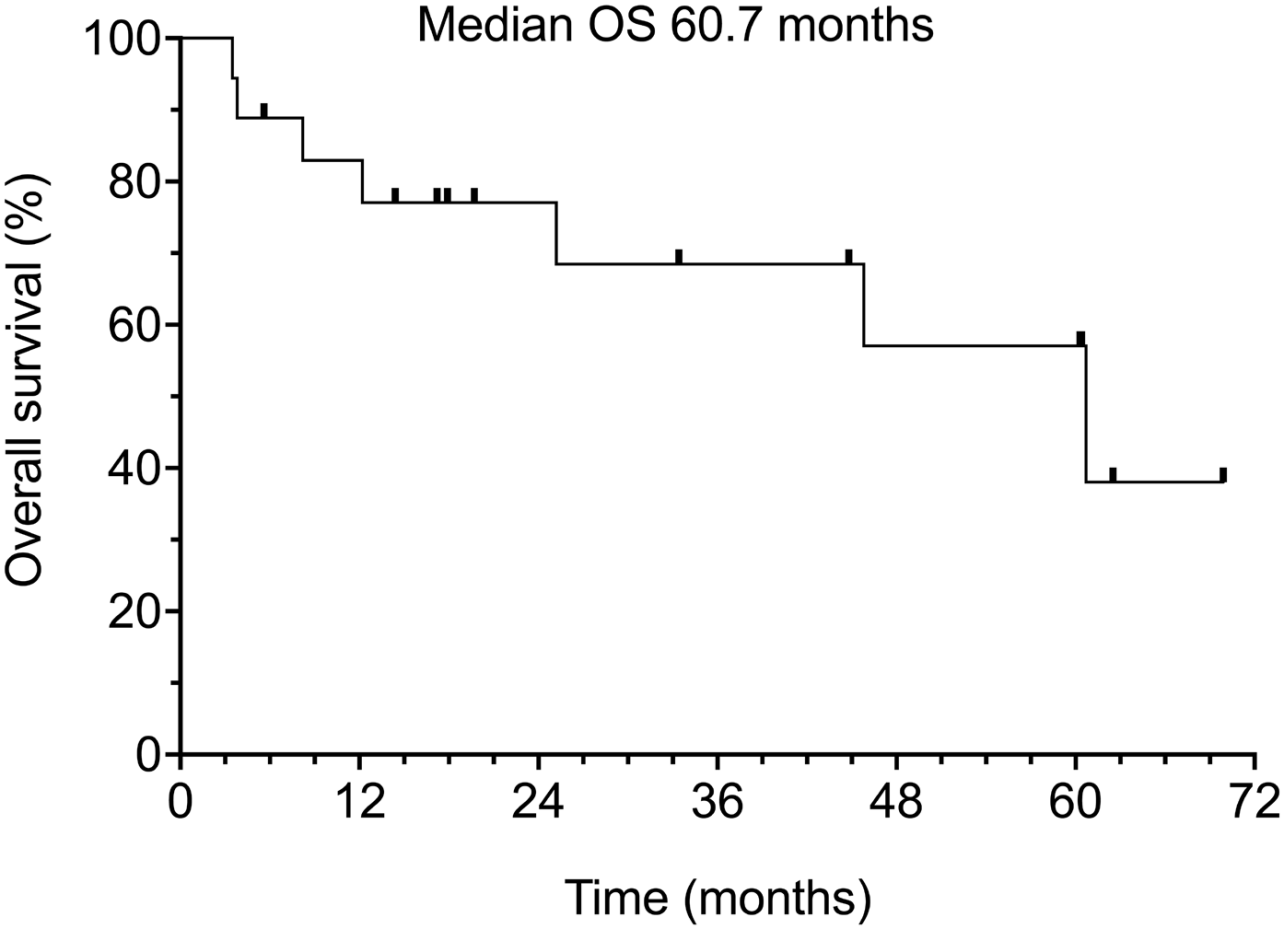
Overall survival of all 18 patients.

Of the 18 patients in our study, 8 (44.4%) have died and 10 (55.5%) remain alive. Median overall survival was 60.7% months (range 3.5–62.5 months) (Figure 3). It should be noted that causes of death included comorbid age-related conditions in 3 patients (16.7%), 1 of whom also had concurrent progression of metastatic CSCC (5.5%), and 3 others developed aggressive secondary neoplasms (16.7%).

Cetuximab treatment was well tolerated. A total of 5 patients experienced no significant cetuximab-related side effects (27.8%). A total of 12 patients (66.7%) developed mild acneiform skin rash controllable with oral and topical antibiotics, or fatigue (reaching at most Grade 1 or 2 toxicity). Radiotherapy produced typical side effects (skin erythema, moist desquamation, and oral mucositis). Three patients had no apparent side effects from radiotherapy (16.7%). Eight patients (44.4%) experienced mild radiation skin changes at the site of radiotherapy. More severe radiation toxicity was seen in other patients, with moist desquamation or other more severe skin toxicity (33.3%), oral cavity mucositis (33.3%), auditory canal inflammation (1 patient). In several patients there were overlapping toxicities. Delayed wound healing at the prior tumor site also was observed after regression of bulky tumors, although this was not precisely quantified. Seven patients required a brief treatment break lasting 1–2 weeks due to radiation dermatitis, moist desquamation, oral pain, and mucositis. Patient number 16 was the only patient to have therapy interrupted more than once for a total of 4 treatment breaks.

Sixteen patients were able to complete planned treatment with minimal side effects. 2 patients (Number 5 and 7) had their treatments discontinued early due to increasing skin toxicity. Despite early discontinuation of therapy in patient 5, after resolution of radiation-induced skin changes, she was found to have achieved a complete response. Patient number 7 developed a second primary cancer, non-Hodgkin’s lymphoma (NHL). This patient was able to restart Erbitux and radiotherapy after being treated with chemotherapy and achieving remission of her NHL. Only 1 patient was ever hospitalized for toxicity. This patient developed syncope due to dehydration while undergoing treatment but was able to continue regimen after recovery (Grade 3 toxicity).

## DISCUSSION

The majority of CSCC are small and localized, and thus are easily managed by dermatologists and surgeons, including MOHS microsurgical approaches. High-risk CSCC are usually managed with aggressive surgical resection including peripheral and deep margin assessment [1]. After resection of high-risk patients, adjuvant radiotherapy is frequently added, based on data from small case series. Unfortunately, no larger or randomized studies of adjuvant radiotherapy have been performed in CSCC, to date. There have been additional recent advances in treatment options for laCSCC such as the PD-1 antibodies pembrolizumab and cemiplimab [17, 18]. Additional treatment options are needed, especially for patients who have contraindications to checkpoint inhibitor treatment, such as those with solid organ transplants and autoimmune disease.

EGFR expression is present in normal keratinocytes and is expressed at increasingly higher levels in most patients with laCSCC and in lymph node metastases from CSCC [12, 20–23]. In keratinocyte cultures, epidermal growth factor (EGF) stimulates cell proliferation and suppresses markers of terminal differentiation [24]. EGF stimulated proliferation can be blocked by EGFR antibodies and EGFR tyrosine kinase inhibitors [24]. *In vitro* and murine models have shown that EGF activation of malignant epithelial cells induces signal transducer and activator of transcription 3 (STAT-3) activation, which drives carcinogenesis [25, 26]. EGFR blockade abrogates this response [25]. Additional preclinical research has shown that EGFR blockade may inhibit telomerase activity in CSCC and thus suppress tumor cell survival [27]. Thus, inhibition of the EGFR signaling pathway seemed to be an attractive treatment option in laCSCC.

Cetuximab is an IgG1 monoclonal antibody, which binds with high affinity to human EGFR. This antibody blocks binding of EGF and other ligands to the EGFR. Cetuximab also induces internalization and downregulation of EGFR and induces enhanced antitumor immune responses via antibody dependent cell-mediated cytotoxicity [28]. *In vitro* exposure of squamous cell carcinoma (SCC) cell lines derived from head and neck cancer patients to cetuximab inhibited proliferation in a time and dose-dependent manner [29]. Since cetuximab causes tumor cells to accumulate in G1 of the cell cycle, radiosensitivity is enhanced [29].

Numerous studies have evaluated cetuximab plus RT in SCCHN. Bonner et al. conducted a randomized trial comparing RT alone with RT plus cetuximab, in the treatment of stage III or IV nonmetastatic, measurable SCC of the oropharynx, hypopharynx, or larynx [30, 31]. The median duration of locoregional control (primary endpoint) was 24.4 months among patients treated with cetuximab plus RT and 14.9 months among those given RT alone. RT plus cetuximab significantly prolonged progression-free survival and overall survival. The 5-year OS rate was 45.6% in the cetuximab plus RT group and 36.4% in the RT alone group [30, 31].

Despite similarities in histology, there has been only limited testing of EGFR antibodies such as cetuximab and panitumumab in CSCC. Published clinical trials of cetuximab and panitumumab in recurrent or refractory CSCC have shown only limited activity as monotherapy [13–16]. Durable responses or remissions have proven elusive [13–16]. The oral 1st generation EGFR inhibitor Erlotinib has demonstrated virtually no clinical activity in CSCC [32].

There is only limited data published concerning the effectiveness of combining cetuximab with radiotherapy in laCSCC. Joseph et al. published 8 patients with laCSCC treated with cetuximab plus concurrent radiotherapy [33]. At 25 months follow-up, five patients remained in a complete remission. One patient relapsed after a partial response. Two patients died (one of due to progression of disease, the other of an unrelated cause). Treatment in this group of patients proved well tolerated, with most toxicities ≤ grade 2, and no toxicities of grade 4/5 reported. Preneau reported a series of patients treated with cetuximab in combination with either radiotherapy, carboplatin or as monotherapy [16]. The response rate in cetuximab and radiation treated patients (*n* = 5) was reported as 80% versus 33% for cetuximab alone. The PFS was a disappointing 1.6 months and overall survival only 3 months for the cetuximab plus radiotherapy patients [16]. Samstein and colleagues reported 12 laCSCC patients with concurrent cetuximab and radiotherapy [34]. Complete and partial response was noted in 36% and 27% (response rate, 64%). Median progression-free and overall survival were only 6.4 and 8.0 months, respectively. Grades 3–4 adverse events were noted in 83% of patients; 67% required hospital admission for adverse events. A total of 51% had longer-term disease control with a short median follow-up (7 months). Lu and Lien published a series of patients receiving either cisplatin and RT (15 patients) or cetuximab plus RT (8 patients) for laCSCC. With 2-year median follow-up, both PFS (50 vs. 30%) and OS (73 vs. 40%) appeared to favor the cetuximab/RT group [35].

The activity of cetuximab therapy in conjunction with radiotherapy in SCCHN encouraged us to use this approach for treatment of laCSCC. This was particularly attractive, as most of our patients had primary tumors originating in head and neck sites. We report 19 patients with laCSCC who had progressed after prior surgery, but who had not received prior systemic therapy or radiation therapy. These patients were treated with a consistent treatment approach combining cetuximab with concurrent radiotherapy. We found an objective response rate of 83.2% with 55.5% complete responses and 27.7% partial responses. The median progression-free survival was 21.6 months, with a median follow-up of 18 months. A total of 60% of patients were progression free at 1 year. Median overall survival was 60.7%.

Our data demonstrate that cetuximab plus radiotherapy represents an active treatment option for laCSCC, with manageable toxicity. This treatment option can be considered in patients with laCSCC, including those with contraindications that preclude checkpoint inhibitor therapy, such as organ transplants and autoimmune disease. The overall frequency of complete responses and duration of responses is high enough to be of interest for potential comparison to checkpoint-inhibitor based therapy. Our current study is intended to be hypothesis generating, with a goal of providing preliminary data to supporting development of comparative clinical trials. Potential limitations of our data include a relatively small number of treated patients, the retrospective nature of the analysis and 6-year span of patient accrual. Challenges for future treatment refinements included a small number of patients who failed either at the treatment site or at the margins of the planned radiation. The latter may suggest expansion of planned treatment volumes. Additional challenges include patients who developed regional and distant metastases from CSCC or aggressive second neoplasms.

## MATERIALS AND METHODS

### Patient population

A retrospective review of medical records was performed of patients uniformly treated with cetuximab by a single physician (WS) at a single institution. These patients were treated over a 6-year period between 2014–2020. Patients with locoregionally advanced or recurrent cutaneous squamous cell skin cancer (CSCC) treated with cetuximab and concomitant radiotherapy were identified by a search of a HIPAA-compliant electronic medical record system, IKnowMed (McKesson, Inc., Houston TX, USA). Clinical data were extracted from the chart into a spreadsheet and de-identified. The following information was extracted from the record: Gender, age, date of diagnosis, primary site, and stage at diagnosis using UICC 8th edition [6]. Characteristics of cetuximab treatment were recorded, including number of doses and cumulative cetuximab dose administered, start and end dates for cetuximab treatment and any cetuximab-associated toxicity. Data related to radiotherapy were also extracted including start and end date of radiotherapy, elapsed time (days), fraction size, cumulative radiotherapy dose (Gy) and any associated toxicities. Best response, date of relapse and progression sites were identified. Both PFS and OS were calculated from cetuximab start date, cause of death was also recorded if patient was deceased. This study design was deemed exempt from full IRB review by the Western IRB chair. The data cut-off date for data analysis was 12/31/20.

Exclusions included patients with squamous cell carcinoma of the head and neck (SCCHN) with oral, pharyngeal, or laryngeal primary sites. Patients with recurrent or metastatic CSCC after prior radiotherapy or other prior systemic treatments or those who were not treated with concurrent cetuximab plus radiotherapy were excluded.

### Cetuximab treatment

The initial loading dose of cetuximab was 400 mg/m² IV infused over 2 hrs. Subsequent doses of 250 mg/m² IV were infused over 60 min each week, starting prior to the initiation of radiotherapy and continuing throughout the entire period of radiation therapy. All patients were seen weekly for close monitoring and lab monitoring during concurrent therapy. Patients were supported with hydration and magnesium replacement as required. Side effects of therapy were treated with symptomatic measures such as topical and oral antibiotics for the frequent cetuximab-induced rash, oral lidocaine for mucositis.

All patients underwent referral and evaluation for concomitant radiation therapy. The treatment consisted of electron beam fields designed to completely encompass superficial skin lesions in most patients, including a 1–2 cm margin. The dose ranged from 5000–6600 cGy at 200–250 cGy per fraction. Field reduction usually after 5000 cGy. If the regional lymph nodes were involved, or the skin tumor invaded facial structures, 3D conformal radiation therapy or IMRT was used with 6MeV photons, bolus used as needed to bring the dose to the surface. For nodal disease, a boost to carry the involved nodes to a total of 6600–7000 cGy was used.

### Data analysis

Extracted information was accessed and recorded in a de-identified manner into a password-protected Excel spreadsheet (Microsoft, Redmond WA) for analysis and calculation of descriptive statistics. Progression-free and overall survival were evaluated via Kaplan-Meier analysis [36]. Toxicity was graded using the CTCAE 1.1 criteria [37].

## Abbreviations

AJCC: American Joint Commission for Cancer
CR: complete remission
CSCC: cutaneous squamous cell carcinoma
EGF: epidermal growth factor
EGFR: epidermal growth factor receptor
laCSCC: locally advanced cutaneous squamous cell carcinoma
NHL: non-Hodgkin lymphoma
PFS: progression-free survival
PR: partial response
SCCHN: squamous cell carcinoma of the head and neck
UICC: Union for International Cancer Control
