# Addendum: Effectiveness and toxicity of cetuximab with concurrent RT in locally advanced cutaneous squamous cell skin cancer: a case series

**DOI:** 10.18632/oncotarget.28480

**Published:** 2023-08-10

**Authors:** Mark Chang, Wolfram Samlowski, Raul Meoz

**Affiliations:** ^1^University Medical Center of Southern Nevada, Las Vegas, NV 89102, USA; ^2^Comprehensive Cancer Centers of Nevada, Las Vegas, NV 89148, USA; ^3^Department of Internal Medicine, University of Nevada, Las Vegas (UNLV), Las Vegas, NV 89102, USA; ^4^Department of Internal Medicine, University of Nevada, Reno, NV 89557, USA

**This article has an addendum:** No external funding was procured for this research.

Original article: Oncotarget. 2023; 14:709–718. 709-718. https://doi.org/10.18632/oncotarget.28470

